# The use of E-cigarettes as a risk factor for oral potentially malignant disorders and oral cancer: a rapid review of clinical evidence

**DOI:** 10.4317/medoral.26042

**Published:** 2023-11-22

**Authors:** Karen Patricia Dominguez Gallagher, Pablo Agustin Vargas, Alan Roger Santos-Silva

**Affiliations:** 1 Oral Diagnosis Department, Semiology and Oral Pathology Areas, Piracicaba Dental School, University of Campinas (UNICAMP), Piracicaba, São Paulo, Brazil; 2 Teaching Assistant Professor, Oral Pathology and Histology, School of Dentistry, National University of Asunción, Paraguay

## Abstract

**Background:**

The popularity of e-cigarettes has increased rapidly in the last decade, particularly among teens and young adults, being advertised as a less harmful alternative to conventional tobacco products. However, *in vitro* and *in vivo* studies have evidenced a variable quantity of potentially harmful components and some recognized carcinogens which may cause DNA damage in oral cells. Additionally, evidence suggests that e-cigarettes may play active roles in the pathogenesis of other malignancies, such as lung and bladder cancers. Therefore, this rapid review aimed to assess the available clinical evidence about using e-cigarettes as a risk factor for oral potentially malignant disorders (OPMD) and oral cancer.

**Material and Methods:**

A systematic search for English language articles published was performed in PubMed (MEDLINE), Embase, Scopus, and Web of Science. After the study selection process, the authors included twelve clinical studies about OPMD and oral cancer risk in e-cigarette users.

**Results:**

The main findings showed the presence of carcinogenic compounds in saliva and morphologic changes, DNA damage, and molecular pathways related to carcinogenesis in the oral cells of e-cigarette users. However, results were inconsistent compared to tobacco smokers and control groups.

**Conclusions:**

the current clinical evidence on this topic is limited and insufficient to support using e-cigarettes as a risk factor for OPMD and oral cancer. Nevertheless, dental care professionals should advise patients responsibly about the potentially harmful effects of e-cigarettes on the oral mucosa cells. Future long-term and well-designed clinical studies are needed.

** Key words:**Oral potentially malignant disorders, oral cancer, e-cigarettes, tobacco.

## Introduction

Oral cancer represents the 18th most common malignancy worldwide, with 377,713 new cases and 177,757 deaths estimated for 2020 (1). In addition, it is considered a significant public health problem because many patients are diagnosed with late-stage disease, which contributes to high morbidity and mortality rates (1,2).

Approximately 90% of malignant tumors affecting the oral cavity are oral squamous cell carcinomas (OSCCs) (3). These neoplasms may arise de novo or be preceded by oral potentially malignant disorders (OPMD), defined as conditions with an increased risk of malignancy (3,4). However, the risk of malignant transformation to carcinoma varies depending on patient- or lesion-related factors (4,5). Tobacco, either in smoked or smokeless forms of consumption, is a well-established risk factor for oral cancer and is associated with an increased risk of malignant transformation of OPMD (3,5).

In the early 2000s, in response to the substantial evidence of cancer risk and general morbidities associated with tobacco consumption, Electronic Nicotine Delivery Systems (ENDS), also referred to as electronic cigarettes (e-cigarettes), e-hookahs, hookah pens, or vapor pens, emerged as safer alternatives to conventional tobacco products or smoking cessation tools and since their commercialization its popularity has been increased among adolescents, and young adults worldwide (6,7). Therefore, the short and long-term effects, including the carcinogenic potential of ENDS, remain an active area of concern and scientific research (7).

There is emerging evidence about the potentially harmful effects of ENDS, including potential oral health consequences related to periodontal tissue, dental problems, oral microbiota alterations, intra-oral explosion injuries, and a variety of symptoms like xerostomia, burning, and irritation observed in e-cigarettes users (6-8). In addition, in vitro and in vivo studies have reported the molecular changes induced by ENDS on the oral cells ranging from reduced anti-oxidant levels and gene dysregulations to DNA strand breaks (9).

Therefore, using e-cigarettes as a harm-reduction tool remains controversial. Many health professionals, including dental clinicians, should provide advice based on scientific evidence, especially to implement preventive measures related to oral cancer and risk factors (8). Thus, this rapid systematic review aims to assess the available clinical evidence about using ENDS as a risk factor for OPMD and oral cancer.

## Material and Methods

Rapid systematic reviews are a type of knowledge synthesis in which components of the systematic review process are simplified or omitted to produce informed evidence and support decision-making in health policy and practice in a short period (10). Following the PRISMA statement, this rapid review was conducted using a systematic review methodology (11) but with some modifications like a shorter search strategy, faster data extraction, and primarily qualitative synthesis. A systematic review protocol was registered in PROSPERO under the registration number CRD42023391709. Despite previous *in vitro* and *in vivo* studies reported DNA damage in oral cells (9) there is still a debate regarding the harmful effects of e-cigarette use which may lead healthcare professionals, including dental practitioners, without confidence when advising patients about using these products. Thus, this rapid review aims to summarize the current clinical findings about using e-cigarettes as a risk factor for OPMD and oral cancer.

- Search strategy

A specific search strategy was designed for this rapid review (Supplement 1). Th e literature search was undertaken on January 13, 2023 in Embase, PubMed, Scopus, and Web of Science. Rayyan Qatar Computing Research Institute (QCRI) software was used to remove duplicate references. In addition, reference lists of included studies were reviewed for citations not captured by the database search.

- Screening and selection of the papers

Considering that there are recently published systematic reviews focused on head and neck cancer risk and cell damage in e-cig users based on detailed analysis of *in vitro* and *in vivo* studies (9,12,13), only clinical evidence was included for this rapid review. We defined clinical evidence as all those studies that included samples from the oral mucosa of electronic cigarette users. The inclusion and exclusion criteria for the study selection are detailed in Table 1. Two reviewers completed the selection in a two-phase process. In phase 1, articles were assessed by title and abstract screening. In phase 2, the studies were selected by full-text reading. In cases of discrepancy, the two authors discussed, and in inconclusive cases, a third author was consulted.

Summary information and characteristics of the included studies were extracted: author and year; country; study design; objectives; methods; sample; main results, and authors' conclusions. Data were extracted by one reviewer and checked by a second reviewer for consistency and correctness. The quality of included studies was assessed using The Joanna Briggs Institute Critical Appraisal Tools (© JBI), and the checklist used varied on the study design. Finally, a narrative synthesis was performed by describing and comparing data reported in the included studies.

**Table 1:**
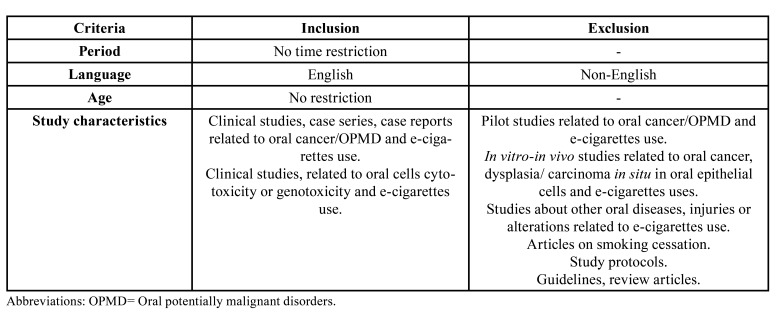
Eligibility criteria considered for study selection process.

## Results

**Figure 1:**
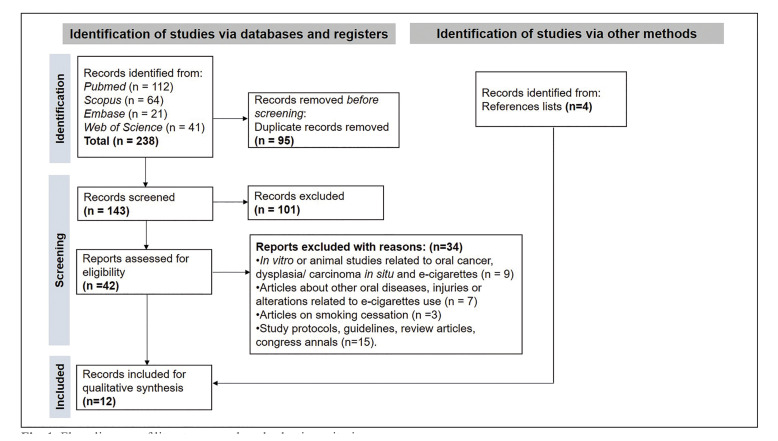
Flow diagram of literature search and selection criteria.

Two-hundred and thirty-eight articles were identified through the electronic literature search. After duplicate remotion, 143 studies were screened by title and abstract, and 101 were excluded. Subsequently, 42 articles were assessed by full-text reading, and 34 records were removed based on exclusion criteria (Supplement 2). Additionally, four studies were identified from the references lists (14-17), all meeting the inclusion criteria. Thus, 12 studies (14-25) were included for qualitative synthesis. The flowchart showing the selection process is detailed in Fig. 1.

Observational studies presenting variable study designs were assessed as clinical evidence regarding using e-cigarettes and OPMD/oral cancer risk. Two were cohort studies (20,21), three were reported cases (14,16,22), and seven were analytical cross-sectional studies (15,17-19,23-25). Among the last group, one article was published as a prospective case-control study (18). Despite including a control group and the sampling being done over two years, data were collected at a single point in time, which is why we classified it as an analytical cross-sectional study.

All included studies were published in English between 2016 and 2022. Six were conducted in the United States (16,19,20-23), two in Italy (15,18), and one each in Brazil (17), Malaysia (25), Romania (24), and the United Kingdom (14).

The risk of bias assessment for the individual studies is summarized in Table 2, and the independently answered questions for each study type are detailed in Supplement 3. Six studies (14,15,18,19,21,23) were classified with moderate risk of bias, four (17,22,24,25) presented with low risk, and two (16,20) with a high risk of bias. The strategies to deal with confounding factors were unclear or not stated in most studies classified with moderate or high risk of bias.

The summarized information of the included articles is detailed in Table 3. In general, nine clinical studies aimed to analyze the carcinogenic effects of e-cigarettes on the oral mucosa. Therefore, the researches were designed as observational studies; most developed a cross-sectional analysis (15,17-19,23-25), and two were conducted as cohort studies (20,21) with 3-6 months of follow-up. However, to describe the current clinical evidence on this topic, we also included reported OPMDs and oral cancer cases identified in ENDS users (14,16,22).

A total of 255 ENDS users were identified among the included studies of this rapid review. The main analyzed materials were the oral cells obtained from mouth brushings and saliva (Table 3). Eight studies (15,17,19-21,23-25) included smokers and non-smokers individuals as comparison groups. An additional group of former smokers was identified in the studies conducted by Schwarzmeier *et al*. (17) and Bardellini *et al*. (18).

Saliva samples were evaluated in two studies (Table 3). First, Bustamante *et al*. 2018 (19) demonstrated the endogenous formation of the carcinogen N´-nitrosonornicotine (NNN) in e-cigarette users. However, compared with smokers, the overall exposure to NNN in e-cigarette users was dramatically lower. Later, Pandarathodiyil *et al*., 2021 (25) reported that the smoker and vaper groups' lactate dehydrogenase (LDH) enzyme levels were significantly higher than in the control group. Nevertheless, no difference in salivary LDH activity level was observed in vapers compared to smokers.

**Table 2:**
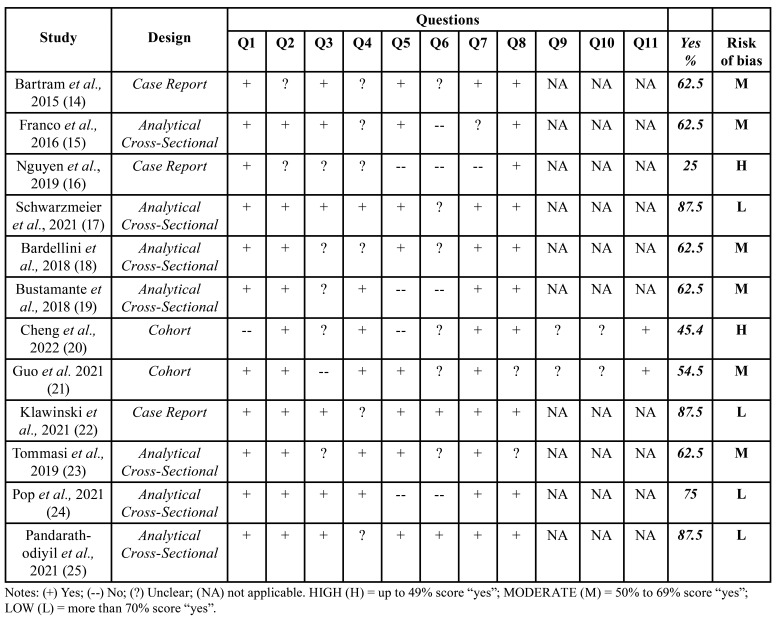
Risk of bias assessed by The Joanna Briggs Institute Critical Appraisal Tools/ © JBI, 2020.

**Table 3:**
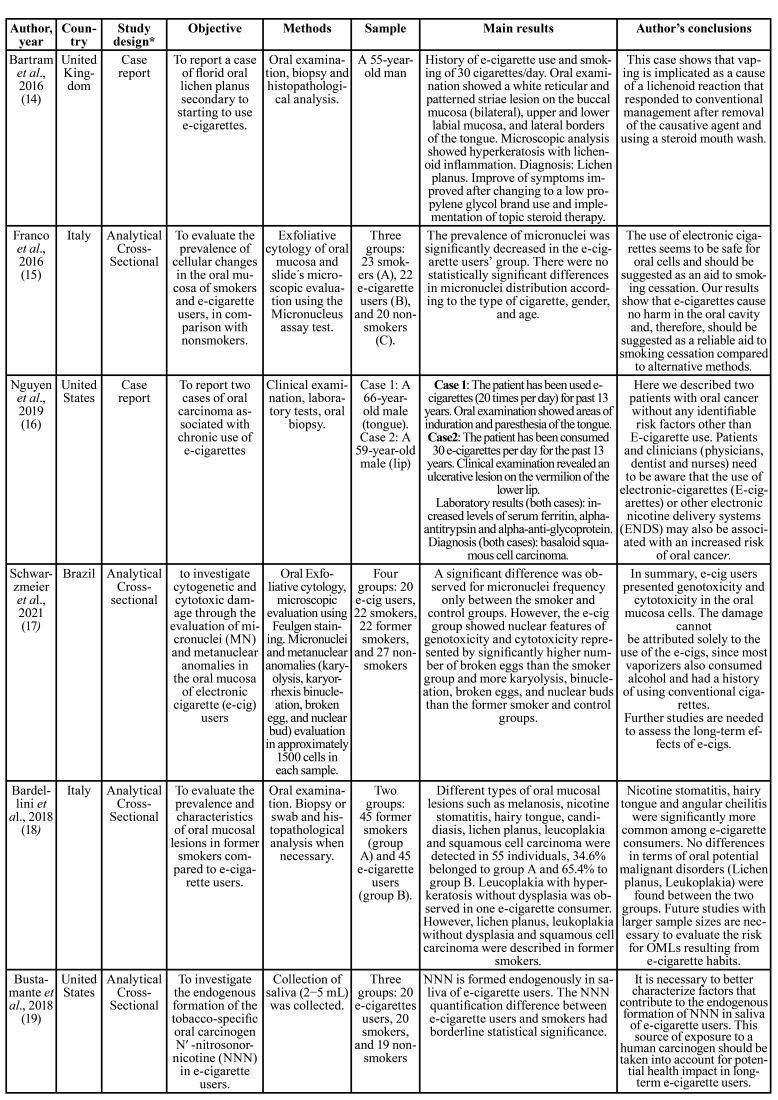
Summarized information, results and conclusions of the included studies (n = 12).

**Table 3 cont.**
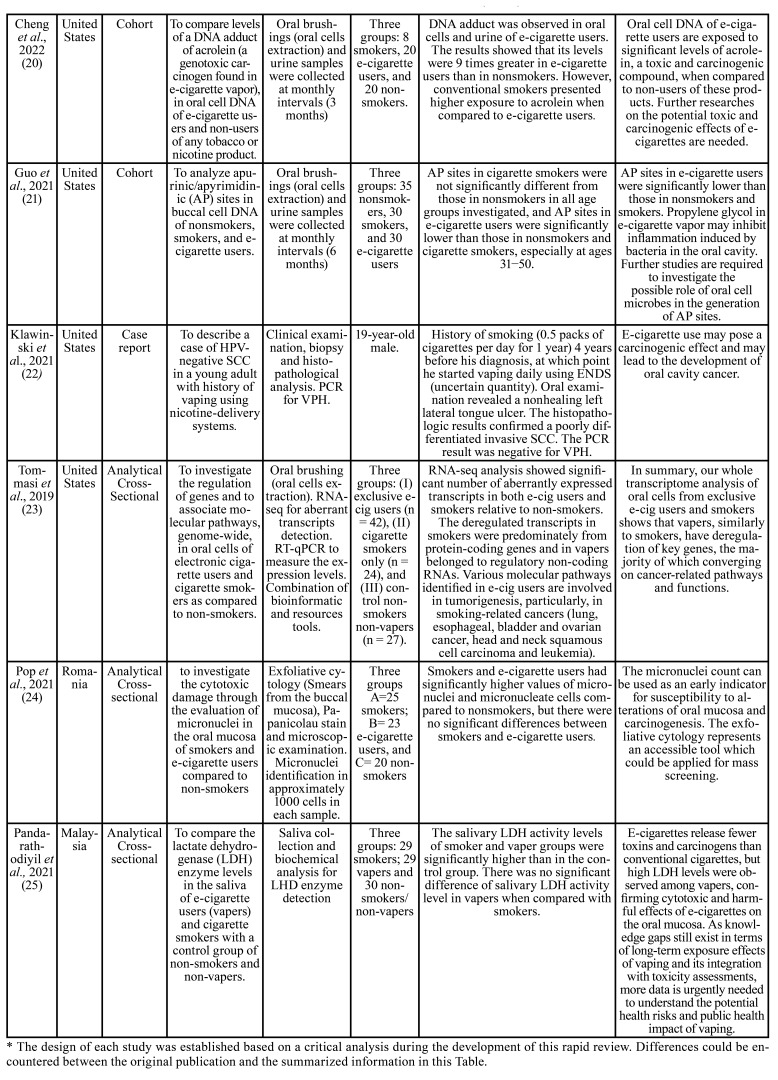
Summarized information, results and conclusions of the included studies (n = 12).

Six studies analyzed the oral cells samples of e-cigarette users (Table 3). Three of them (15,17,24) used the micronucleus assay test through microscopic evaluation of exfoliative cytology samples for analyzing the carcinogenic effects of e-cigarette exposure in oral cells. Franco *et al*. 2016 (15) identified a low prevalence of micronuclei in the e-cigarette users’ group compared to smokers and described that these values were similar to nonsmokers, so they suggested that using ENDS seems to be safe for oral cells. In contrast, Pop *et al*., 2021 (24) found significantly higher values of micronuclei in e-cigarette users and smokers compared to nonsmokers. However, they reported no significant differences between smokers and e-cigarette users. In the study by Schwarzmeier *et al*., 2021 (17), besides the micronuclei assay, they also evaluated the metanuclear anomalies of oral cells among e-cigarette users and compared results between smokers, former smokers, and nonsmokers. These study results showed a significant difference in micronuclei frequency between smokers and nonsmokers. However, the e-cigarette group showed more metanuclear anomalies than the smoker, former smoker, and control groups suggesting the genotoxicity and cytotoxicity in the oral mucosa cells of ENDS users.

The reminded three studies (20-23) evaluated the DNA damage of oral cells and suggested the potential genotoxicity caused by e-cigarette exposure. The study conducted by Cheng *et al*. 2022 (20) showed higher levels of DNA adduct of acrolein in e-cigarette users than non-smokers. However, smokers presented higher exposure to acrolein when compared to e-cigarette users. Guo *et al*. 2021 (21) analyzed the DNA exposure to mutagens in oral cells of vapers, smokers, and non-smokers based on identifying apurinic/apyrimidinic (AP) sites. They described that AP sites in e-cigarette users were significantly lower than in smokers and non-smokers and stated that propylene glycol in e-cigarettes might inhibit bacteria in the oral cavity, resulting in reduced inflammation and related effects, and consequently reduced AP site levels in e-cigarette users' DNA. Finally, Tommasi *et al*. 2019 (23), through RNA-seq analysis, showed increased numbers of deregulated transcripts in vapers. Additionally, the bioinformatic analysis identified that various molecular pathways presented in e-cigarette users are responsible for tumorigenesis in smoking-related cancers.

Regarding OPMD in ENDS users, we identified one case report (14) of oral lichenoid reaction in a 55-year-old man with a history of e-cigarette use. According to this report, the white-striated lesions coincidently appeared when he started using an e-cigarette. Furthermore, the clinic-pathological correlation stated the diagnosis of lichenoid eruption related to e-cigarette use, which improved after removing the causative agent (e-cigarette) and using a steroid mouthwash (Table 3). An analytical cross-sectional study by Bardellini *et al*. 2018 (18) aimed to describe the prevalence of oral lesions in e-cigarette and former smokers. They described various types of lesions in both groups and among e-cigarette users, only one patient presented leucoplakia (hyperkeratosis without dysplasia). In contrast, the oral lesions in former smokers included two oral lichen planus, two leucoplakias without dysplasia, and one OSCC.

There are also reported cases of oral cancer in e-cigarette users (Table 3). For example, in 2019, Nguyen *et al*. (16) reported two basaloid squamous cell carcinoma cases in two male adults without any identifiable risk factors other than e-cigarette use. One of these cases affected the tongue, and the other, the lower lip. Later, in 2021, Klawinski *et al*. (22) described a case of HPV- negative OSCC in a young adult with a history of vaping. However, the patient mentioned a previous history of smoking (0.5 packs of cigarettes per day for one year) before using e-cigarettes. In both articles, the authors concluded that there might be a carcinogenic effect in e-cigarettes which may represent an increased risk of oral cancer.

## Discussion

Considering the increasing global use of ENDS, most of the current research is focused on the role of e-cigarettes in cancer pathogenesis. Growing evidence suggests that they may play active roles in lung and bladder cancers (26,27). Therefore, several *in vivo* and *in vitro* studies are conducted to understand their short- and long-term effects on oral health, including oral carcinogenesis. The results described so far DNA damage in the oral cells of e-cigarette users (9). However, there is limited clinical evidence about e-cigarette use as a potential risk for OPMD and oral cancer. Thus, this rapid review assessed the published clinical studies in English and summarized the available data about ENDS users' risk for developing of OPMD or oral cancer.

Most clinical studies included in this rapid review were designed as observational studies with cross-sectional analysis to understand the potential oral carcinogenic effects of e-cigarettes. However, to consider e-cigarettes an independent causative factor for a multifactorial disease like oral cancer, it is essential to identify the presence of confounding factors to make a reliable inference (28). Unfortunately, our results showed that most studies presented a moderate risk of bias due to unclear or negative answers regarding identifying or managing confounding factors. Hence, alcohol consumption, type, quantity, time of e-cigarette use, and the fact that e-cigarettes are mainly used by tobacco and former smokers (3,5,17,29) are essential confounding factors to consider in future studies. The remaining articles included were cohort studies with limited exposure time and small sample size, as well as case reports, which led to only low-level evidence (30) could be included in this rapid review.

The studies described potential carcinogenic effects in ENDS users' saliva and oral cells. For example, high levels of the carcinogen NNN, LDH enzyme, and DNA adduct of acrolein (19,20,25) and increased numbers of deregulated transcripts and molecular pathways involved in tumorigenesis of smoking-related cancers were identified in e-cigarette users (23). However, these findings are inconsistent when compared with smokers and controls. In addition, three studies on micronuclei evidenced contradictory results when comparing e-cigarette users with non-users (15,17,24). One of them also reported more metanuclear anomalies in vapers suggesting its genotoxicity and cytotoxicity (17).

Nevertheless, one study described the DNA damage in oral cells measured by AP sites and reported significantly lower levels in e-cigarette users than in smokers and non-smokers (21). Therefore, based on the mentioned findings, the available data might be critically analyzed before discussing with patients the "safeness" or "benefits" of e-cigarette use. First, however, it is essential to point out evidence of potential damage and risks related to its use due to the proven or potential carcinogenic agents identified in e-cigarettes components, including metals (cadmium, chromium, etc.), carbonyls (acrolein), propylene oxide, and especially flavoring additives (9,28).

Few studies have characterized ENDS-related oral mucosal lesions and reported xerostomia, nicotine stomatitis, hairy tongue, angular cheilitis intra-oral explosion injuries, periodontal disease, and oral microbiome alterations were frequently found in e-cigarette users (7). However, there is scarce evidence about OPMD and oral cancer in ENDS users. We found a reported case of oral lichenoid eruption associated with e-cigarette use (14) and a cross-sectional study describing one case of oral leukoplakia without dysplasia among 45 e-cigarette users (18). Despite this, it is essential to highlight that lacking long-term clinical studies on using e-cigarettes leads to a scarcity of direct evidence for association of e-cigarettes with oral malignant transformation. However, persistent local irritation may result in pathological mucosal reactions that may predispose to the development of OPMD (28).

Regarding oral cancer, there are 3 cases reported in the literature, and the presence of vaping history is a common finding in all cases (16,22). Nevertheless, isolated reported cases are not considered strong enough for a causal relationship between oral cancer and e-cigarette use. However, they help to generate a hypothesis for causal inference for future studies (26).

It is important to highlight some limitations of this review, like the exclusion of non-English primary articles during the literature search, which might lead to possible missed relevant publications. Additionally, the included studies presented different designs. Most were conducted without property identification of confounding variables such as type, time, and concentrations of e-cigarette liquid or vapor, exclusive e-cigarette use, or combination with conventional forms of tobacco and alcohol consumption. The sample selection and analysis variation among the cited studies is also a limitation.

## Conclusions

In summary, there is limited and low-quality clinical evidence associating using ENDS with malignant transformation for OPMD and oral carcinogenesis. Therefore, it might be too soon to include e-cigarette use as a risk factor for OPMD and oral cancer. However, based on current findings, clinicians and dental-care professionals must carefully advise patients about the use of e-cigarettes especially considering that even lower dosages of potential carcinogens present in e-cigarettes, compared to conventional tobacco smokers, could induce molecular changes in the oral mucosa and DNA damage in oral cells with no apparent clinical change. Future research on this topic is needed especially long-term studies with larger sample sizes, including individuals that are exclusively e-cigarette users and considering that newer generations of e-cigarettes with different designs and patterns are being produced.

## References

[1] Sung H, Ferlay J, Siegel RL, Laversanne M, Soerjomataram I, Jemal A (2021). Global Cancer Statistics 2020: GLOBOCAN Estimates of Incidence and Mortality Worldwide for 36 Cancers in 185 Countries. CA Cancer J Clin.

[2] Warnakulasuriya S, Kerr AR (2021). Oral Cancer Screening: Past, Present, and Future. J Dent Res.

[3] Miranda-Filho A, Bray F (2020). Global patterns and trends in cancers of the lip, tongue and mouth. Oral Oncol.

[4] Warnakulasuriya S (2020). Oral potentially malignant disorders: A comprehensive review on clinical aspects and management. Oral Oncol.

[5] Speight PM, Khurram SA, Kujan O (2018). Oral potentially malignant disorders: risk of progression to malignancy. Oral Surg Oral Med Oral Pathol Oral Radiol.

[6] Esteban-Lopez M, Perry MD, Garbinski LD, Manevski M, Andre M, Ceyhan Y (2022). Health effects and known pathology associated with the use of E-cigarettes. Toxicol Rep.

[7] Sultan AS, Jessri M, Farah CS (2021). Electronic nicotine delivery systems: Oral health implications and oral cancer risk. J Oral Pathol Med.

[8] Briggs K, Bell C, Breik O (2021). What should every dental health professional know about electronic cigarettes?. Aust Dent J.

[9] Guo J, Hecht SS (2022). DNA damage in human oral cells induced by use of e-cigarettes. Drug Test Anal.

[10] Tricco AC, Antony J, Zarin W, Strifler L, Ghassemi M, Ivory J (2015). A scoping review of rapid review methods. BMC Med.

[11] Page MJ, McKenzie JE, Bossuyt PM, Boutron I, Hoffmann TC, Mulrow CD (2021). The PRISMA 2020 statement: An updated guideline for reporting systematic reviews. BMJ.

[12] Wilson C, Tellez Freitas CM, Awan KH, Ajdaharian J, Geiler J, Thirucenthilvelan P (2022). Adverse effects of E-cigarettes on head, neck, and oral cells: A systematic review. J Oral Pathol Med.

[13] Szukalska M, Szyfter K, Florek E, Rodrigo JP, Rinaldo A, Mäkitie AA (2020). Electronic Cigarettes and Head and Neck Cancer Risk-Current State of Art. Cancers (Basel).

[14] Bartram A, Jones N, Endersby S (2016). Lichenoid eruption associated with use of an e-cigarette. Br J Oral Maxillofac Surg.

[15] Franco T, Trapasso S, Puzzo L, Allegra E (2016). Electronic Cigarette: Role in the Primary Prevention of Oral Cavity Cancer. Clin Med Insights Ear Nose Throat.

[16] Nguyen H, Kitzmiller JP, Nguyen KT, Nguyen CD, Bui TC (2017). Oral Carcinoma Associated with Chronic Use of Electronic Cigarettes. Otolaryngol.

[17] Schwarzmeier LÂT, da Cruz BS, Ferreira CCP, Carvalho BFDC, Alves MGO, Lima Carta CF (2021). E-cig might cause cell damage of oral mucosa. Oral Surg Oral Med Oral Pathol Oral Radiol.

[18] Bardellini E, Amadori F, Conti G, Majorana A (2018). Oral mucosal lesions in electronic cigarettes consumers versus former smokers. Acta Odontol Scand.

[19] Bustamante G, Ma B, Yakovlev G, Yershova K, Le C, Jensen J (2018). Presence of the Carcinogen N'-Nitrosonornicotine in Saliva of E-cigarette Users. Chem Res Toxicol.

[20] Cheng G, Guo J, Carmella SG, Lindgren B, Ikuemonisan J, Niesen B (2022). Increased acrolein-DNA adducts in buccal brushings of e-cigarette users. Carcinogenesis.

[21] Guo J, Ikuemonisan J, Hatsukami DK, Hecht SS (2021). Liquid Chromatography-Nanoelectrospray Ionization-High-Resolution Tandem Mass Spectrometry Analysis of Apurinic/Apyrimidinic Sites in Oral Cell DNA of Cigarette Smokers, e-Cigarette Users, and Nonsmokers. Chem Res Toxicol.

[22] Klawinski D, Hanna I, Breslin NK, Katzenstein HM, Indelicato DJ (2021). Vaping the Venom: Oral Cavity Cancer in a Young Adult with Extensive Electronic Cigarette Use. Pediatrics.

[23] Tommasi S, Caliri AW, Caceres A, Moreno DE, Li M, Chen Y (2019). Deregulation of Biologically Significant Genes and Associated Molecular Pathways in the Oral Epithelium of Electronic Cigarette Users. Int J Mol Sci.

[24] Pop AM, Coroș R, Stoica AM, Monea M (2021). Early Diagnosis of Oral Mucosal Alterations in Smokers and E-Cigarette Users Based on Micronuclei Count: A Cross-Sectional Study among Dental Students. Int J Environ Res Public Health.

[25] Pandarathodiyil AK, Ramanathan A, Garg R, Doss JG, Abd Rahman FB, Ghani WMN (2021). Lactate Dehydrogenase Levels in the Saliva of Cigarette and E-Cigarette Smokers (Vapers): A Comparative Analysis. Asian Pac J Cancer Prev.

[26] Lee HW, Park SH, Weng MW, Wang HT, Huang WC, Lepor H (2018). E-cigarette smoke damages DNA and reduces repair activity in mouse lung, heart, and bladder as well as in human lung and bladder cells. Proc Natl Acad Sci U S A.

[27] Zahedi A, Phandthong R, Chaili A, Remark G, Talbot P (2018). Epithelial-to-mesenchymal transition of A549 lung cancer cells exposed to electronic cigarettes. Lung Cancer.

[28] Raj AT, Sujatha G, Muruganandhan J, Kumar SS, Bharkavi SI, Varadarajan S (2020). Reviewing the oral carcinogenic potential of E-cigarettes using the Bradford Hill criteria of causation. Transl Cancer Res.

[29] Martins BNFL, Normando AGC, Rodrigues-Fernandes CI, Wagner VP, Kowalski LP, Marques SS (2022). Global frequency and epidemiological profile of electronic cigarette users: a systematic review. Oral Surg Oral Med Oral Pathol Oral Radiol.

[30] Arieta-Miranda JM, Ruiz-Yasuda CC, Pérez Vargas LF, Torres Ricse DA, Díaz SP, Arieta YC (2022). New Pyramid Proposal for the Levels of Scientific Evidence According to SIGN. Plast Reconstr Surg.

